# The epidemiology of rape and sexual violence in the platinum mining district of Rustenburg, South Africa: Prevalence, and factors associated with sexual violence

**DOI:** 10.1371/journal.pone.0216449

**Published:** 2019-07-31

**Authors:** Sarah Jane Steele, Naeemah Abrahams, Kristal Duncan, Nataly Woollett, Bella Hwang, Lucy O’Connell, Gilles van Cutsem, Amir Shroufi

**Affiliations:** 1 Médecins Sans Frontières (MSF) South Africa, Operational Control Centre Brussels, Cape Town, South Africa; 2 South African Medical Research Council, Gender and Health Division, Cape Town, South Africa; 3 University of the Witwatersrand, School of Clinical Medicine, School of Public Health, Johannesburg, South Africa; 4 Médecins Sans Frontières (MSF), Southern African Medical Unit, Cape Town, South Africa; 5 University of Cape Town, Centre for Infection Disease and Epidemiology Research, Cape Town, South Africa; Stellenbosch University, SOUTH AFRICA

## Abstract

**Background:**

Estimates for the prevalence of rape and other forms of sexual violence (SV) vary in South Africa. This survey aimed to provide clarity by quantifying the prevalence of SV (forced sex or sexual acts) by 1) sexual partners, and 2) non-partners, and to describe factors associated with these outcomes among women (18–49 years) living in Rustenburg Municipality.

**Materials and methods:**

We conducted a cluster-randomized household survey (November—December 2015). Women were asked about their experiences of SV, associated attitudes and behaviours, and access to services. Logistic regression was used to determine factors associated with partner and non-partner SV.

**Results:**

Of eligible households, 83·1% (1700/2044) participated. Of 966 women invited, 836 participated (86·5%). Average age of participants was 31.6 years (95%CI: 30·9, 32·4) with 45% having completed at least secondary school, and 60% unemployed or looking for work.

Lifetime prevalence of SV was 24.9% (95%CI: 21·7–28·5), reaching 9.0% (95% CI: 6·6–12·1) by age 15. Almost one third told no one of their SV experiences. Factors related to financial dependence were associated with SV by a partner. History of termination of pregnancy increased the likelihood of SV by a non-partner as an adult. Women who experienced SV in childhood or as an adult were more likely to experience SV from a different type of perpetrator than those who did not.

**Conclusions:**

We found a high prevalence of SV, including during childhood, in this setting, with limited access to care. This and the high morbidity attributed to SV calls for increased service provision.

## Introduction

Sexual violence (SV), including rape, is a human rights violation with extensive health impacts.[1] Rape forms part of a broad range of SV acts and refers to forced penetrative sex within the South Africa law[2]. Although a growing body of literature on violence against women and children has emerged in the last 10 years, the focus has been on the prevalence and drivers of intimate partner violence, commonly combining physical and sexual violence.

Rape and other forced sexual acts by partners and non-partners have received very little focused research attention. Globally, there are limited estimates of partner rape. This is largely due to the difficulty of measuring a stigmatized experience with most studies focusing on broad concepts of SV. The WHO estimates that 35.6% of women have ever experienced either partner physical or sexual violence or non-partner sexual violence (with estimates for the African region (36.6%) being among the highest)[3]. Global prevalence of SV, including rape and other forced sexual acts by non-partners is estimated at 7.2% (95%CI: 5.2% - 9.1%)[4], with the prevalence in Southern Africa estimated at 17.4% (95%CI: 11.4–23.3%)[4].

Estimates of rape prevalence in South Africa vary widely within and across provinces, with rape by a partner ranging from 1·5% to 18·8% [5–8] and non-partner rape ranging from 2·1% to 12·2%[6,9]. This may be due to actual differences across locations and/or an artifact of differences in the measures used. Uncertainty as to how many people in South Africa are affected means that gaps in terms of service access, the adequacy of existing service capacity and coverage, as well as those at particular risk of rape and other forms of SV, are hard to assess. In South Africa experience of rape and other forms of SV are associated with the education and income levels of both the survivor and the perpetrator, the characteristics of and equality within the intimate partnership, and societal norms around patriarchy and violence.[10]

SV, in general, and rape specifically lead to both mental and physical health problems–including unintended pregnancy,[11–13] substance and alcohol use, and several psychological distress/ psychiatric morbidities, such as depression, post-traumatic stress disorder and suicide.[14] These sequelae increase risk behavior and other vulnerabilities, including staying in abusive intimate relationships, which place women and their children at risk of violence in the future.[10] Sexual violence and rape are also associated with the acquisition of STIs[15,16] including HIV.[17,18] The latter being of particular importance in South Africa, where there are concurrent HIV and SV epidemics.[19] Despite this, few women seek medical care or psycho-social care.[20]

In Rustenburg, a fast growing city in South Africa’s North West province, high levels of immigration driven by the lure of work in the mining sector[21] create a social environment in which rape and other forms of sexual violence may be particularly prevalent.[22–24] Médecins Sans Frontières (MSF), in collaboration with the Department of Health, provides free provision of comprehensive medical care, and counselling for survivors of sexual violence through both a vertical (stand-alone) sexual violence center as well as a mentorship program in providing care for rape survivors for nurses and other service providers at primary health care centers. This study provides information regarding the epidemic of SV that can be used to help frame a response to it. Findings from this research, including both prevalence as well as factors associated with SV, will inform service provision and support regional as well as national advocacy work for more comprehensive and accessible service provision. The specific objectives of this analysis were to quantify the prevalence of rape and SV by 1) sexual partners, and 2) non-partners, and to describe factors associated with these outcomes among women (aged 18–49 years) living in Rustenburg Municipality, South Africa.

## Methods

### Overview of study design and sampling

We conducted a cluster-randomized household survey in Rustenburg Municipality, in the Bonjanala Health District of North West Province, South Africa (November—December 2015) in line with standard practice and WHO methodology, safety and ethical guidelines.[1,25]

We used a multi-stage systematic sampling procedure. To ensure self-weighting the first two stages were sampled proportionate to population size. The first cluster was census main place. Among 66 census main places in Rustenburg Municipality 61 were included. Main places with small population sizes were grouped together, resulting in 26 primary sampling units (PSUs) being sampled out of a total of 34. Two PSUs were excluded due to logistical considerations, therefore the final number of PSUs included was 24. The second cluster was census small area. Among 674 census small areas in the included main places, 156 were sampled and 120 were ultimately included (after the two PSUs were excluded). The sampling frame utilized geographic units and data (2011) from Statistics South Africa. The included areas represented 141,491 of the total 179, 543 eligible population in Rustenburg Municipality (78.8%).

We aimed to include a fixed number of houses (12) from within each small area. To allow for replacement (e.g., for buildings that were not houses or which were destroyed) we sampled 24 houses within each area. Houses were visited up to three times after which no further visits were made. We applied realization weights to small areas to account for the fact that we did not achieve 12 eligible houses in each area. The realization weight was equal to 12 divided by the total number of houses included from each area and ensured equal representation of each included small area[26,27]. Within each selected household all women aged 18–49 living in Rustenburg Municipality (for at least 4 weeks prior to screening were considered eligible, with the participant chosen randomly where more than one eligible woman was identified.

Study staff received an eight day training on research methods and on issues of sexual and gender-based violence prior to the start of the survey. To insure staff safety interviewers worked in pairs. One team leader led two interviewer pairs. Written, informed consent was obtained from all participants. Appropriate referral to services (with transport support) was carried out after each interview.[25]

### Measurement

Data were collected on tablet computers using the CommCare platform (Dimagi, Inc) via face-to-face interviews (conducted in English, Setswana, isiXhosa, Afrikaans, or Tsonga) and uploaded daily to a central electronic database.

To ensure the reliability and validity of our results, we used normative measures and definitions.[1,5] Rape was defined as having experienced forced sex (Table 1). SV was defined as having experienced forced sexual or sexual acts. Lifetime prevalence of SV was calculated as the number of women who had at least one experience of SV with either a partner or a non-partner. We utilized a broad definition of sexual partner which included sexual partners to whom the participant was married, living with as if married or had a regular ongoing relationship with. We collected detailed information on the participants’ current or most recent partner, including experiences of recent (i.e. in the previous 12 months) and non-recent SV as well as other forms of emotional, physical and sexual intimate-partner violence. All variables related to physical and sexual violence were measured on a 4-point scale never, once, a few times and many times. This was later dichotomized for analysis (Never/ At least once).

**Table 1:** Definitions of rape and sexual violence and methods of measurement.

Variable	Definition	Measurement
**Rape, Current or most recent partner**	“*physically forced you to have sexual intercourse when you did not want to*” with current or most recent husband/partner	All women who indicated that they had ever been in an intimate partnership (i.e. been married, lived with a man as if married, had an ongoing regular sexual relationship) were included in the analysis of rape.
**Sexual violence, all current and/or previous partners~**	“physically forced you to have sexual intercourse when you did not want to” with current or most recent husband/partner AND/OR “*physically forced to have sexual intercourse or perform sexual acts that she did not want to*” with any other partner	All women who indicated that they had ever been in an intimate partnership (i.e. been married, lived with a man as if married, had an ongoing regular sexual relationship) were included in the analysis of partner-SV.
**Sexual violence, non-partner**	“*forced to have sexual intercourse or perform sexual acts that she did not want to*” with someone whom she was not in an intimate partnership at the time of the incident	Participants were asked to describe the experiences of non-partner rape with different types of perpetrators before (child) and since the age of 15 years (adult). This division between adult and childhood experiences of rape was chosen to facilitate comparisons with other studies using the WHO Multi-Country study methodology

We also collected information about socio-demographics, current/most recent partner characteristics, sexual and reproductive history, and attitudes and community norms (S1 Appendix).

### Statistical analysis

Analyses were conducted in STATA13. Point estimates were weighted to account for the selection probability of the cluster sampling procedure. Standard errors and 95% confidence intervals were calculated using the Taylor linearization method and single sample strata (i.e., strata with only one sampling unit represented) were centered at the overall mean [27]. Logistic regression was used to assess variables associated with SV. Post-estimation procedures were used to calculate the adjusted prevalence differences and adjusted prevalence ratios[28]. Variable selection procedures for regression models were consistent with published recommendations for prediction models [29,30]. While these models are not meant to be used for prediction per se, this analytic process was chosen because it is able to highlight variables most relevant to sexual violence within each theoretical grouping. A best-subsets approach to modeling was utilized[29]. To summarize, crude bivariable models were used to assess associations between selected variables and both 1) partner sexual violence, and 2) non-partner sexual violence. Variables that were significant at the p<0.25 level were candidates for subset analyses. Final models were selected using a backwards selection procedure. Variables were removed from the final models starting with the least significant. The exit criteria was p<0.30 and the entry criteria was p<0.25, with consideration of significance of individual levels of a variable as well as goodness-of-fit of the model. The backwards selection procedure with a forwards elimination continued until all variables that remained in the model met entry (p<0.25) and exit (p < .30) criteria. Overall model fit was assessed using Pearson goodness of fit tests. Subsets were determined based on a review of the literature and framed around an eco-social framework[31–34]. The ecosocial framework was used to structure ideas and conceptualize the way in which social and biological processes are embodied and manifest in the epidemiologic profiles of women living in Rustenburg Municipality[35,36]. Subsets investigated included: socio-demographics; partnership; reproductive history; sexual history; attitudes and community norms; consequences of sexual violence. Details of the variable in each subset are included inAppendix A.

### Ethics

Ethics approval was obtained from the University of Cape Town Human Research Ethics Committee (HREC REF: 799/2014) and the MSF Ethics Review Board (ID: 1538). The research project was granted approval by the Research Office of North West Province Department of Health.

## Results

**Fig 1:**
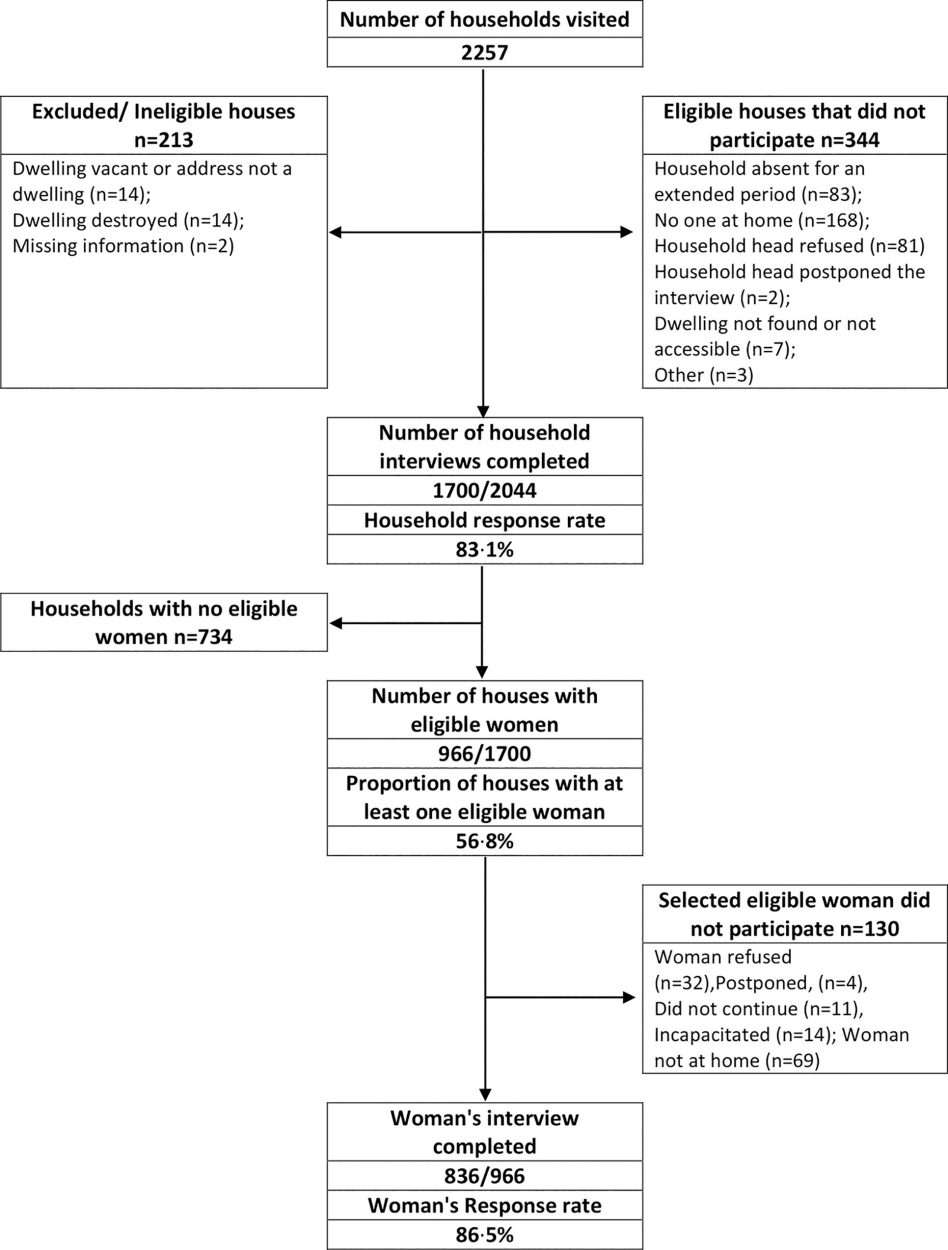
Flowchart of Rustenburg Women’s Health Survey, a cluster-randomised household survey conducted in Rustenburg Municipality, 2015.

Of 2257 households visited, 2044 were eligible and 1700 household interviews were completed (Fig 1, household response rate: 83·1%). A large number of houses did not have eligible women. Of 966 households with at least one eligible woman, 836 of the randomly selected women participated (Woman’s response rate: 86·5%). Women who indicated that they never had sex (n = 31) were not asked questions regarding forced sex and other experiences of sexual violence with partners and non-partners. As some women may not have considered experiences of forced sex and other types of sexual violence has intercourse we excluded these women from the analysis.

The average age of participants was 31.6 years (95%CI: 30·9, 32·4) (Table 2). The majority of women, 97.2% (95%CI: 95·5–98·3) were literate, and almost half completed at least secondary school. Almost two-thirds were unemployed or looking for work and 42·6% (95%CI: 38·6–46·8) indicated they would not be able to raise sufficient money to support themselves and their family for four weeks. Approximately 35% of women were currently married or living with a man as if they were married, and an additional 42·0% had a regular sexual partner. Approximately, 80% of women had ever given birth.

**Table 2:** Characteristics of women who participated in the Rustenburg Women’s Health Survey conducted in Rustenburg Municipality, 2015.

		Mean		95% Confidence interval	
				Lower limit	Upper limit
**Age**		31·6		30·9	32·4
**Age of sexual initiation**		17.8		17·6	18·0
		n*	**%**		
**Place of birth (N = 825)**	Rustenburg	279	50·7	42·7	58·7
	South Africa, Outside of Rustenburg	358	40·3	33·5	47·4
	Outside of South Africa	76	7·5	5·2	10·8
	Unclassified	12	1·5	0·7	3·0
**Education (N = 829)**	No schooling or Adult-based education	18	1·8	1·1	3·2
	At least some primary	100	10·7	8·6	13·1
	Started secondary	350	42·1	3·8	46·1
	Completed secondary	267	33·9	3·0	37·8
	At least some post-secondary	94	11·5	9·3	14·1
**Employment (N = 828)**	Unemployed / looking for work	488	60·0	54·7	65·0
	Blue collar/ Trade/ Manual labour/ Other	192	21·6	18·0	25·7
	Student	57	8·8	6·7	11·6
	Professional/ White collar	54	6·0	4·2	8·6
	Housewife	37	3·6	2·5	2·5
**Owns a house** **(N = 828)**	No	524	68·5	61·9	74·4
	With others	167	17·8	14·1	22·3
	On her own	137	13·7	10·4	17·7
**Owns large house hold items^ (N = 824)**	No	296	41·1	35·8	46·7
	With others	186	19·6	16·1	23·6
	On her own	342	39·3	34·9	43·9
**Unable to support oneself and one's family for 4 weeks in an emergency (N = 814)**		324	42·6	38·6	46·8
**Partnership status (N = 827)**	Currently married	211	20·6	17·9	23·5
	Living with a man as if married, but not married	159	14·0	11·8	16·4
	Regular sexual partner, not living together	299	42·0	38·1	46·1
	Not currently in a sexual relationship	158	23·5	20·0	27·3
**Reproductive history** **(N = 827)**	Never given birth or been pregnant	125	18·8	15·0	23·2
	Never had a TOP~	665	72·0	72·0	80·7
	Ever had a TOP~	37	4·6	3·1	6·8

*Unweighted

^Large household items, for example TV, bed, cooker

~TOP: Termination of pregnancy

Lifetime prevalence of SV was 24·9% (Table 3). Among women who had ever experienced SV, 32% (95%CI: 24·2–42·1) had experienced SV by both a partner and non-partner, 41·0% (95%CI: 32·7–49·9; n = 87) by a non-partner only, and 26·5% (95%CI: 19·9–34·4; n = 54) by a partner only. Among women who had experienced SV since the age of 15 years, 30·6%(95%CI: 22·7–39·9; n = 50) had experienced SV by both a partner and non-partner (as an adult), 32·8% (; 95%CI: 24·9–41·8; n = 64) by a non-partner only (as an adult), and 36·6% (95%CI: 28·1–46·0; n = 62) by a partner only.

**Table 3:** Prevalence and frequency of rape and other forms of sexual violence among women who participated in the Rustenburg Women’s Health Survey conducted in Rustenburg Municipality, 2015 (Weighted estimates).

**Prevalence**	**Childhood, <15 years**				**Adulthood, ≥ 15 years**				**Lifetime**			
	n*	**%**	**95% CI**		n*	**%**	**95% CI**		n*	**%**	**95% CI**	
			**LL**	**UL**			**LL**	**UL**			**LL**	**UL**
**Sexual violence**												
**Sexual violence,** all current and/or previous partners~					112/744	16·0	13·3	19·2				
**Sexual violence**, non-partner	66/798	9·0	6·6	12·1	114/798	13·9	11·2	17·1	145/798	18·3	15·4	21·7
**Sexual violence**, partner and or non-partners									199/798	24·9	21·7	28·5
**Rape**												
**Rape**, current/ most recent partner~					64/745	9·4	7·3	12·1				
**Frequency**	n*	**%**	**95% CI**		n*	**%**	**95% CI**		n*	**%**	**95% CI**	
			**LL**	**UL**			**UL**	**LL**			**LL**	**UL**
**Sexual violence,** with non-partners, (N = 796)												
More than three times	4	0·5	0·1	1·4	16	2·1	1·2	3·7				
Two or three times	8	0·7	0·3	1·6	33	3·4	2·2	5·2				
Once	54	7·8	5·6	10·8	65	8·4	6·3	11·1				
Zero	730	91·0	87·9	93·4	682	86·1	82·9	88·8				
**Rape**, in the previous 12 months with intimate partners~, (N = 747)												
Yes, many times					19	2·82	1·71	4·6				
Yes, a few times					24	2·8	1·76	4·5				
Yes, once					18	2·63	1·43	4·8				
Yes, but more than 12 months ago					51	7·7	5·6	10·5				
Never					635	84·0	80·9	86·8				

*Unweighted

~Among ever partnered women

Eight percent of ever partnered woman experienced SV within the previous 12 months. Among women who had ever experienced partner SV, 51·7% (95%CI: 40·3–63·0) had experienced it at least once in the previous 12 months.

Variables associated with having experienced partner and non-partner SV are given in Table 4. Goodness of fit tests were not significant suggesting adequate overall model fit. There was a decreased likelihood of reporting partner sexual violence (Adjusted prevalence difference (APD) -0·08, 95%CI: -0·13–0·02) among women who were able to support themselves and their families for one month compared to women who were not able to support themselves. There was a 12 percentage point increase (APD: 0·12, 95%CI: 0·05–0·20) in the likelihood of experiencing partner SV among women whose husband/ partner had ever refused to give them money for household expenses compared to women whose husbands/ partner had never refused. A strong positive association was also found with physical violence from an intimate partner (APD: 0·19: 8·6,95%CI: 0·14–0·24). Associations with non-partner sexual violence as an adult showed an increased likelihood of experiencing partner SV (APD: 0·27, 95%CI: 0·17–0·36). Positive associations with non-partner SV were also observed with knowing one other woman who experienced sexual violence (APD: 0·07, 95%CI: 0·01–0·12), and among women who indicated that giving woman food in exchange for sex was SV (APD: 0·11, 95%CI: 0·06–0·16).

**Table 4:** Variables associated with partner (N = 716) and non-partner sexual violence (N = 728) since the age of 15 years.

		Partner rape						Non-partner rape (≥ 15 years)					
		Un-adjusted Prev	95% CI		Adjusted Prev	95% CI		Un-adjusted Prev	95% CI		Adjusted Prev	95% CI	
			Lower limit	Upper limit		Lower limit	Upper limit		Lower limit	Upper limit		Lower limit	Upper limit
**Sociodemographics**													
**Age**	18 years (Ref)	9·81%	5·34%	14·28%	8·99%	5·43%	12·54%						
	49 years	26·85%	17·19%	36·52%	28·07%	20·97%	35·17%						
	**Prevalence diff**	0·17	0·04	0·30	0·19	0·10	0·28						
	**Prevalence ratio**	2·74	1·29	5·78	3·12	1·79	5·45						
	Odds ratio*	1·04	1·01	1·07	1·07	1·03	1·11						
**Able to support oneself and one's family for 4 weeks in an emergency**	No (Ref)	0·20	0·15	0·25	0·21	0·16	0·26						
	Yes	0·14	0·11	0·18	0·13	0·11	0·16						
	**Prevalence diff**	-0·05	-0·12	0·01	-0·08	-0·13	-0·02						
	**Prevalence ratio**	0·73	0·51	1·05	0·64	0·47	0·87						
	**Odds ratio**	0·68	0·42	1·11	0·44	0·24	0·82						
**Reproductive history**													
**History of TOP**~	Never TOP~ (Ref)							13·59%	10·78%	16·40%	13·86%	10·87%	16·86%
	Never given birth or been pregnant							9·47%	3·65%	15·30%	12·79%	6·20%	19·38%
	**Prevalence diff**							-0·04	-0·10	0·02	-0·01	-0·08	0·06
	**Prevalence ratio**							0·70	0·38	1·29	0·92	0·54	1·56
	**Odds ratio**							0·67	0·31	1·42	0·87	0·31	2·40
	TOP~							33·07%	15·98%	50·16%	25·83%	13·52%	38·14%
	**Prevalence diff**							0·19	0·02	0·36	0·12	0·00	0·24
	**Prevalence ratio**							2·43	1·43	4·15	1·86	1·17	2·97
	**Odds ratio**							3·14	1·33	7·44	3·13	1·14	8·55
**Partnership characteristics**													
**Husband had ever refused to give money for household expenses**	No (Ref)	12·58%	9·68%	15·49%	13·72%	10·86%	16·58%						
	Yes	38·29%	28·15%	48·44%	26·07%	18·99%	33·16%						
	**Prevalence diff**	0·26	0·15	0·37	0·12	0·05	0·20						
	**Prevalence ratio**	0·86	0·22	3·46	1·90	1·36	2·65						
	**Odds ratio**	4·31	2·39	7·78	3·24	1·63	6·47						
	N/A he had no savings or earnings to share	10·86%	-4·03%	25·75%	16·86%	2·59%	31·13%						
	**Prevalence diff**	-0·02	-0·17	0·13	0·03	-0·11	0·17						
	**Prevalence ratio**	0·86	0·22	3·46	1·23	0·52	2·89						
	**Odds ratio**	0·85	0·15	4·71	1·42	0·27	7·45						
**Experienced physical violence from an intimate partner**	No (Ref)	4·23%	2·01%	6·45%	5·69%	2·94%	8·45%						
	Yes	29·05%	24·15%	33·95%	24·62%	20·47%	28·77%						
	**Prevalence diff**	0·25	0·19	0·30	0·19	0·14	0·24						
	**Prevalence ratio**	6·87	3·90	12·09	4·33	2·62	7·15						
	**Odds ratio**	9·27	4·69	18·29	8·60	4·45	16·60						
**Sexual history**													
**First sexual experience**	By choice							9·04%	6·33%	11·76%	10·31%	7·20%	13·43%
	Did not want to but was not forced							11·52%	6·32%	16·73%	14·13%	8·88%	19·39%
	**Prevalence diff**							0·02	-0·03	0·08	0·04	-0·02	0·10
	**Prevalence ratio**							1·27	0·75	2·18	1·37	0·86	2·18
	**Odds ratio**							1·31	0·68	2·54	1·58	0·75	3·34
	Forced							0·66	0·54	0·78	0·45	0·30	0·60
	**Prevalence diff**							0·57	0·44	0·69	0·35	0·19	0·50
	**Prevalence ratio**							7·27	5·10	10·36	4·35	2·74	6·89
	**Odds ratio**							19·30	9·57	38·91	11·92	4·67	30·43
**Experienced NP~~ Rape as an adult (> = 15 years)**	No (Ref)	10·27%	7·75%	12·79%	11·13%	8·42%	13·84%						
	Yes	52·17%	41·68%	62·66%	37·83%	28·96%	46·71%						
	**Prevalence diff**	0·42	0·31	0·53	0·27	0·17	0·36						
	**Prevalence ratio**	5·08	3·67	7·04	3·40	2·39	4·84						
	**Odds ratio**	9·53	5·40	16·82	8·63	4·42	16·83						
**Experienced rape as a child**	No (Ref)							10·27%	7·85%	12·69%	31·82%	18·90%	44·75%
	Yes							50·61%	36·36%	64·86%	12·25%	9·55%	14·95%
	**Prevalence diff**							0·40	0·26	0·54	0·20	0·07	0·32
	**Prevalence ratio**							4·93	3·53	6·88	2·60	1·71	3·95
	**Odds ratio**							8·96	4·71	17·03	5·22	2·15	12·64
**Experience rape by a partner (> = 15 years)**	No (Ref)							9·48%	6·89%	12·07%	10·12%	7·52%	12·73%
	Yes							25·96%	19·28%	32·63%	32·51%	22·59%	42·44%
	**Prevalence diff**							2·74	1·86	4·03	0·22	0·13	0·32
	**Prevalence ratio**							0·16	0·09	0·24	3·21	2·22	4·64
	**Odds ratio**							9·53	5·40	16·82	6·42	3·15	13·10
**Attitudes and community norms**													
**Know any women who have experienced SV**^	No (Ref)	11·64%	9·03%	14·24%	13·83%	10·96%	16·71%	9·48%	6·89%	12·07%	12·29%	9·06%	15·51%
	Yes	26·51%	19·97%	33·05%	20·47%	15·70%	25·25%	25·96%	19·28%	32·63%	18·34%	13·07%	23·61%
	**Prevalence diff**	2·28	1·62	3·20	0·07	0·01	0·12	0·16	0·09	0·24	0·06	0·00	0·12
	**Prevalence ratio**	0·15	0·08	0·22	1·48	1·11	1·98	2·74	1·86	4·03	1·49	1·02	2·17
	**Odds ratio**	2·74	0·00	1·71	4·40	1·11	3·66	3·35	1·98	5·64	1·99	1·02	3·88
**Giving a woman food in exchange for sex is SV**^	No (Ref)	6·88%	2·82%	10·94%	7·07%	2·80%	11·35%						
	Yes	18·03%	14·90%	21·16%	17·92%	14·99%	20·84%						
	**Prevalence diff**	0·11	0·06	0·16	0·11	0·06	0·16						
	**Prevalence ratio**	2·62	1·42	4·84	2·53	1·39	4·61						
	Odds ratio*	2·98	1·43	6·20	4·52	1·74	11·76						

~~Non-partner

~Termination of pregnancy

^Sexual violence

*Age treated as a continuous variable

Women who reported ever having had terminated a pregnancy were 12 percentage points more likely (95%CI: 0·00–0·24) () to report non-partner SV as an adult (>15 years of age) than those who had not. Women who experienced SV as a child were 20 percentage points more likely (95%CI: 0·07–0·32) to report non-partner SV as an adult and an even stronger association was found among those whose first sexual experience was forced (APD: 0·35, 95%CI: 0·19–0·50) compared to women whose first sexual experience was by choice.

The likelihood of non-partner SV as an adult was 22 percentage points higher (APD: 0·22, 95%CI: 0·13–0·32) among women who experienced partner SV than among those who did not experience partner SV. Participants who knew any other women who had experienced SV were more likely to experience non-partner SV as an adult than those who did not know any women who had experienced SV.

Among women who experienced both partner and non-partner SV, disclosure of the experiences of SV were similar– 30·1% (95%: 21·2–40·9) of women who experienced partner SV and 28·0% (95%CI: 18·8–39·6) of women who experienced non-partner SV did not tell anyone. Approximately 90% of women did not report to police and 95% did not report to a medical or psycho-social service provider.

## Discussion

Prevalence of SV, during childhood and adulthood, is high among women living in Rustenburg, South Africa. As most women did not seek medical care after experiences of rape and other forms of SV, the opportunity to address the medical and psycho-social consequences is not realized in most cases.

Our data suggest that 1 in 4 women in the Rustenburg area have experienced SV in their lifetime, many frequently. Nine percent of women experienced SV before the age of 15 years. Globally SV by an intimate partner is more common than SV by a non-partner.[37] In our study SV by a non-partner in adulthood (13·9%; 95%CI: 11·2, 17·1) was almost as common as SV by a partner (16·0%; 95%CI: 13·3–19·2). This may reflect that women are less likely to identify SV by a partner as SV[38] or that SV by non-partners is more common than in other areas. These findings are in line with that found in Gauteng in 2010[6], but also suggest that the prevalence of SV in Rustenburg is at least double what has been reported in other areas of South Africa[8,39]. It is also higher than estimates for Southern Africa (17·4%; 95%CI: 11·4–23·3%) and globally (7·2%; 95%CI: 5.2% - 9.1%)[4]. This may be related to the atypical social dynamics of Rustenburg, which is characterized by a high proportion of male only households in informal settlements, often inhabited by those working in blue collar mining jobs and whom have migrated from other areas of South Africa—in South Africa men who are relatively advantaged and whom have some degree of power, but live in poor communities are more likely to perpetrate rape.[40]

We found that women in Rustenburg experience multiple incidents of SV alongside other forms of violence throughout their lifetime. For example, the experience of SV in childhood or as an adult was associated with increased likelihood of SV from a different type of perpetrator. This clustering of experiences may reflect intrinsic vulnerabilities as well as the environmental and social characteristics of the area.

We found little to link attitudes related to intimate partnerships or SV with experiences of SV. The exception was the finding that women who had experienced SV by a partner were 11 percentage points more likely to indicate that giving a woman food in exchange for sex is a form a SV. We also found that women whose husbands had ever refused to give money for household expenses increased the likelihood of partner SV while the ability to support oneself and one’s family in an emergency was protective. This suggests that financial dependence within intimate partnerships, real and perceived, may be a stronger vulnerability in this population than other attitudes and norms. The lack of financial autonomy among survivors of SV highlights the importance of ensuring that survivors can access services without incurring costs to themselves.

Post-traumatic stress disorder (PTSD)[19], depression,[1,41,42] and substance use disorders[42] are well known psychological sequelae of rape and other forms of SV. These negative mental health consequences can contribute to the intrinsic vulnerabilities that place an individual at future risk of rape.[10] Through similar pathways, children who experience violence or witness their mother experience violence are more likely to experience or perpetrate sexual violence as adults[8] contributing to the cycle of intergenerational risk. While appropriate counseling has been shown to mitigate or eliminate the effects of PTSD, depression and other morbidities, a recent assessment in South Africa found poor integration of mental health services into post-rape care and a general lack of capacity to provide appropriate and compassionate acute and long-term mental health services.[43] Women who do not receive mental health services will continue to suffer unduly from mental health morbidities and may also continue a cycle of vulnerability and risk.

While the gaps in protocol and access for the provision of mental health services have been noted in South Africa,[43] the medical protocols for survivors of rape and SV are clear and specific. Understanding the scope of rape and SV helps to understand how much of health system resources should be focused specifically on providing care to rape survivors–including the provision of accessible post-exposure prophylaxis, emergency contraception and termination of pregnancy (TOP).

We observed an association between non-partner sexual violence and TOP. This may be because of an increased likelihood of seeking TOP due to the SV or it may be because of confounding factors related to both outcomes. This association again points to a clustering of vulnerability and trauma among women in the Rustenburg area. Although globally, evidence suggests an association between intimate partner violence and both unintended pregnancy, and TOP[11,12] we did not find evidence of an association between partner SV and TOP. In this analysis we did not measure unintended pregnancy and it is possible that non-partner sexual violence is associated with unintended pregnancy but that women in established partnerships do not necessarily seek to end these. None-the-less, intimate partner violence and unintended pregnancy can lead to an intergenerational and repeated cycle of unintended pregnancies and intimate partner violence at individual and community levels.[12] Services and screening protocols for TOP, intimate partner violence and SV should also be integrated in order to open access pathways across services and avoid the secondary trauma of seeking services more than once. Currently, as few women access services, the opportunity for emergency contraception is lost.

In our study most of those who had been raped or whom had experienced SV did not report to police or seek medical care. There are a range of barriers to reporting sexual violence to the police in South Africa. These include: feelings of shame and self-blame; societal attitudes and discrimination against those who have been victims of SV; community taboos around SV; reluctance towards or threats against reporting a family member or intimate partner; belief that the perpetrator will not be punished, and; discriminatory attitudes by police and other legal officials[20]. Reasons for failure to report to medical services include a lack of awareness of the availability of such services, lack of knowledge of the preventability of diseases such as HIV, fear of stigma[44] that may be caused by rape and other forms of SV or the mistaken belief that one has to report to the police in order to access medical care. While most women did not report to formal services, the majority did tell someone of their experiences, most frequently friends and family. This suggests that opportunities exist to improve access to services not just through health promotion activities targeting survivors but also through those close to them.

Despite using validated and standard measures, we relied on self-report in face-to-face interviews. Social desirability and stigma may result in under-reporting of experiences of rape and other forms of violence. In line with methodological and safety guidelines for research about sexual violence, this study only included women. We acknowledge that this misses important information about perpetration by and victimization of men. In this area, where there are large numbers of men, future research should quantify the prevalence of victimization as well as perpetration in order to inform prevention programming and service provision.

We had a high response rate and relatively large sample size. Despite this, we still had small sample sizes in some strata limiting the precision of our estimates. We utilized a modeling approach which favoured the strongest predictors, which means there is a possibility that some relevant variables were excluded from the final models. This cross-sectional study cannot not determine temporality or causality. Given the nature of the study, we cannot determine whether or not the sexual violence occurred in Rustenburg. However, we found a clustering of risk and vulnerability, including multiple experiences of sexual violence with different types of partners, high levels of unemployment, and financial instability. Access to sexual violence services, in particular those for mental health, are important for women regardless when or where the incident took place.

The number of population-based studies which specifically assess the prevalence of rape and SV in South Africa are limited, dated and show variability in estimates. No study has been conducted in the unique context of the platinum mining belt. This study was conducted among a representative sample of women living in Rustenburg. It generates new knowledge and complements the existing body of literature, strengthening evidence on the high prevalence of rape and sexual violence. While not generalizable to all of South Africa our findings are likely similar to the experiences of women living in fast growing areas characterized by a large number of dispersed settlements, large proportions of internal migrants and a high male to female ratio.

## Conclusions

We report an extremely high prevalence of SV, including during childhood, in the platinum belt of Rustenburg. This includes rape by partners and non-partners, making women vulnerable to mental and physical trauma, and HIV acquisition. In South Africa there is a coordinated HIV response; however, a focus on HIV prevention and justice, alongside the failure to approve a sexual assault policy, have left gaps in the provision of mental health and medical services for survivors of rape and other forms of SV. A patient-centered response to rape should link legal, medical and psycho-social services at primary health care level to ensure access for women living in dispersed settlements such as in the platinum mining belt and those living in other rural and peri-urban areas.

## Supporting information

S1 Appendix Measurement of factors collected and tested to determine associations with partner and non-partner sexual violence since the age of 15 years. (DOC)

S1 Dataset Dataset. (DTA)
