# Supplementary material for: The epidemiology of rape and sexual violence in the platinum mining district of Rustenburg, South Africa: Prevalence, and factors associated with sexual violence
Source: PLoS One. 2019 Jul 31;14(7):e0216449. doi: 10.1371/journal.pone.0216449 (PMC6668777; doi:10.1371/journal.pone.0216449)
Supplement: S1 Appendix — (DOC) [file pone.0216449.s001.doc]

## Appendix A:

| **Appendix A: Measurement of factors collected and tested to determine associations with partner and non-partner sexual violence since the age of 15 years.** | | |
| --- | --- | --- |
| **Subset** | **Variable** | **Measurement** |
| **Socio-demographics** | **Age** | Age was collected as a continuous variable and treated as continuous for analysis. Women who did not know their age were asked to provide their date of birth which was then used to determine age at the time of the survey. |
|  | **Place of birth and migration history** | Participants were asked if they were born and raised in Rustenburg and if not they were asked to indicate where they were born and raised using opened-ended questions. Responses for birth place were then categorized for analysis as: Rustenburg; South Africa, Outside of Rustenburg; Outside of South Africa. Responses which could not be categorized were indicated as “Unclassified”. |
|  | **Education** | Education was collected as a categorical variable: No schooling; Adult-based education; started primary; Completed primary; Started secondary; Completed secondary, and; At least some post-Secondary. These categories were subsequently collapsed for analysis as follows: No schooling or adult-based education; At least some primary; Started secondary; Completed secondary, and; At least some post secondary. |
|  | **Employment** | Participants were asked about their profession and interviewers categorized the responses as: Unemployed/ Looking for work; Blue collar (Mining/ Non-Mining); White collar (Mining/ Non-mining); Trade/Business; Manual labour; Professional, Student, Housewife, Retired, Seasonal Work and; Other. |
|  | **Financial** | To assess participants’ ability to support themselves in case of an emergency, participants were asked to indicate (Yes/No) whether or not they alone could raise enough money to house and feed their family for four weeks. |
|  |  | The participants were asked about whether or not they owned ("*Yes, own by self*;" "*Yes, own with others*;" "*No, don't know*") each of the following items: Land; Your house; Any other properties; a company or business; large animals; small animals, large household items; Jewelry, gold or other valuables; Responses were dichotomized (Yes/No) for analysis and explored both individually and as a count of the total number of items owned. |
| **Partnership** | **Access to household earnings** | Access to household earnings was determined using two questions: “*How often has your husband/ partner taken your earnings against your will*” and “*How often has your husband/ partner refused to give you money for household expenses even when has many for other things.*” Both were collected on a 4-point likert scale and dichotomized for analysis. |
|  | **Controlling behaviours** | Participants were asked (Yes/No) if their current or most recent partner had ever done any of the following: *"He (is/was) jealous or angry if you (talk/talked) to other men*; "*He frequently (accuses/accused) you of being unfaithful;*" "*He (does/did) not allow you to meet your female friends*;" *He (tries/tried) to limit your contact with your family;*" "*He (insists/insisted) on knowing where you (are/were) at all times?*" During analysis this was assessed as a count of the number of controlling behaviours and also as a dichotomous variable (None/ At least one). |
|  | **Emotional intimate partner violence** | Participants were asked how often (Never/Once/A few times/ Many times/ Refused) they had experienced each of the following with their current or most recent partner had: "*… said or done something to humiliate you in front of others;*" "*…threatened to hurt or harm you or someone you care about*," "*...insulted you or made you feel bad about yourself.*" During analysis this was assessed as a count of the number of behaviours and also as a dichotomous variable (None/ At least one). |
|  | **Physical intimate partner violence** | Participants were asked how often (Never/Once/A few times/ Many times/ Refused) they had experienced each of the following with their current or most recent partner had: "…slapped or thrown something at you that could hurt you," "pushed you or shoved you," "hit you with his fist or something else that could hurt you;" "...kicked you, dragged you, or beaten you," "...choked or burnt you on purpose;" "...threatened to use or actually used a gun, knife or other weapon against you." Similarly, participants were asked if they had experienced these behaviours from any other partners. During analysis this was assessed as a count and also as a dichotomous variable (None/ At least one). |
|  | **Sexual intimate partner violence (Current or most recent)** | Participants were asked how often (Never/Once/A few times/ Many times/ Refused) they had experienced each of the following with their current or most recent partner had: "physically forced you to have sexual intercourse when you did not want to"; "had sexual intercourse with your current or most recent husband/partner when you did not want because you were afraid of what he might do" and "forced you to do something sexual that you found degrading or humiliating". For the purpose of analysis this was dichotomized as never/ at least once. Being "physically forced to have sexual intercourse when you did not want to," was rape was treated separately from other forms of sexual violence from curren't most recent partner. |
|  | **Partner sexual violence (any partner) (Primary outcome)** | “*Physically forced to have sexual intercourse when you did not want to”* with the current or most recent partner AND/*OR “physically forced to have sexual intercourse or perform any sexual acts when you did not want to”* by another sexual partner. |
| **Reproductive history** | **Reproductive history** | Participants were asked whether or not they had ever given birth (yes/no) and whether or not they had ever been pregnant (yes/no/maybe/unsure). These questions were validated alongside the number of pregnancies, number of live births, number of miscarriage and number of terminations to determine if they woman had ever been pregnant and the number of children she had had. |
|  | **Paternity of children** | Participants were asked if all of their children had the same biological father and were given the following response options: "Yes, one father;" "No, more than one father;" "I don't know". This was treated as an independent, categorical variable in the analysis. |
|  | **History of termination of pregnancy** | Participants were asked if they had ever used anything or tried in any way to terminate or end an existing pregnancy (Yes/No). |
| **Sexual history** | **Age at sexual debut** | Participants were asked how old they were when they first had sex. Age in years was collected as a continuous variable. During analysis age at sexual debut was explored as a continuous variable and also as a categorical variable. Ultimately, it was treated as a continuous variable |
|  | **Experience of sexual debut** | Participants were asked to indicate how the perceived their first sexual experience: "*You wanted to do it*;" :*You did not want to but were not forced*;" "*You were forced to do it.*" This was treated as an independent categorical variable in the analysis. |
|  | **Transactional sex** | Participants were read the following statement: "*Some people receive money, gifts, or other goods in exchange for sex*" and asked to indicate whether or not (Yes/No) they had ever, now or in the past, had sex for money, food or other gifts. |
| **Attitudes and community norms** | **Attitudes about acceptable behaviour within a relationships** | Participants were asked if they agreed or disagreed with each of the following statements: "*A good wife obeys her husband even if she disagrees*;" "*Family problems should only be discussed with people in the family;*" "*It is a wife's obligation to have sex with her husband even if she does not feel like it;*" "*If a man mistreats his wife, others outside the family should intervene;*" "*If a man beats his wife, others outside the family should intervene;*" "*If a man verbally abuses his wife, others outside the family should intervene.*" During analysis these were explored independently and as count of the total number statements that a participant agreed with. |
|  | **Attitudes about relationships between men and women** | Participants were asked to indicate (Yes/No) their opinion on each of the following statements: "*Does a man have a good reason to hit his wife if she does not complete her household work to his satisfaction*;" "*Does a man have a good reason to his wife if she disobeys him*;" "*Does a man have a good reason to his wife if she refuses to have sexual relations with him*;" "*Does a man have a good reason to hit his wife if she asks him whether he has other girlfriends;*" "*Does a man have a good reason to hit his wife if she has been unfaithful*." During analysis these were explored independently and as count of the total number statements that a participant agreed with. |
|  | **Attitudes about refusal of sex within a marriage** | Participants were asked about their opinion (yes/no) on the following statements: "*Can a married woman refuse to have sex with her husband if he is drunk*;" "*Can a married woman refuse to have sex with her husband if he is drunk*;" "*Can a married woman refuse to have sex with her husband if she is sick*;" *Can a married woman refuse to have sex with her husband if he mistreats her.*" During analysis these were explored independently and as count of the total number statements that a participant agreed with. |
|  | **Attitudes about what constitutes sexual violence** | Participants were asked about their opinions (yes/no) regarding what acts constitute sexual violence. "*Is a woman being forced by her husband to have sexual intercourse an act of sexual violence*;" "*Is a woman being touched inappropriately by an older family member an act of sexual violence;*" "*Is giving food to a woman in exchange for sex an act of sexual violence;*" "*Is being sexually assaulted by a stranger or group of strangers an act of sexual violence;*" "*Is the sexual assault of a child an act of sexual violence.*" During analysis these were explored independently. |
|  | **Attitudes about who can experience sexual violence** | Participants were asked about whether they agreed or disagreed with each of the following statements: "*Women can experience sexual violence*;" "*Children can experience sexual violence*," and "*Men can experience sexual violence.*" During analysis these were explored independently. |
|  | **Beliefs about the apportioning of blame** | Participants were asked about their opinions about the frequency (Always/ Never/ Sometimes/ Never/ Not applicable, a woman cannot experience sexual violence) in which sexual violence is the fault of the survivor. Participants were asked the following questions with respect to female (women), male (men), and child survivors: "*Yes it is always a [woman's/man's/child's] fault;*" "*Most of the time it is a [woman's/man's/child's] fault;*" *"Sometimes it is a [woman's/man's/child's] fault;*" "*No, it never a [woman's/man's/child's] fault;*" "*No, it is never a [woman's/man's/child's] fault,*" and; "*Not applicable, a [woman/man/child] cannot experience sexual violence.*" During analysis these were explored independently. |
|  | **Awareness of people who have experienced sexual violence** | Women were asked *“Do you personally know any women in your community who have experienced sexual violence?*”; *“Do you personally know any men in your community who have experienced sexual violence?*”; “*Do you personally know any children in your community who have experienced sexual violence?*” The answers to each of these were dichotomous and each variable was assessed separately at both the univariable and multivariable stages of modeling. |
| **Consequences of sexual violence** | **Consequences of sexual violence on mental health** | Participants were asked whether or not the agreed or disagreed with each of the following statements: "*Sexual violence can have a negative impact on the mental wellbeing of the person who experienced it;*" "*Sexual violence can make someone withdraw from their friends and family;*" "*Sexual violence can make someone depressed;*" "*Sexual violence can make someone suicidal,*" and "*Sexual violence can affect someone's self-esteem.*" During analysis these were explored independently and as a count of the total number statements that a participant agreed with. |
|  | **Consequences fo sexual violence on stigma** | Participants were asked whether they agreed or disagreed with the following statements (don't know was an option) related to stigma: "*Sexual violence can lead to stigma;" "People might avoid someone who has experienced sexual violence," and; "People might call someone who has experienced sexual violence names."* During analysis these were explored independently and as a count of the total number statements that a participant agreed with. |
|  | **Consequences of sexual violence on physical health** | Participants were asked about consequences of rape as an act of forced vaginal or anal penetration. They were asked to agree or disagree (don't know was an option) with the following statements: "*Rape can lead to HIV infection;*" "*Rape can lead to sexually transmitted infections (STIs);*" "*Rape can lead to pregnancy;*" "*Someone can be injured from being raped;*" "*Someone can die from being raped.*" During analysis these were explored independently and as a count of the total number of statements that a participant agreed with. |
|  | **Knowledge of post rape medical treatment** | Participants were asked about different medical services available after rape. They were asked to agree or disagree (don't know was an option) with the following statements: "*Rape can lead to HIV infection*;" "*Rape can lead to sexual transmitted infections (STIs)*;" "*Rape can lead to pregnancy;*" "*Someone can be injured from being raped;*" "*Pregnancy can be prevented after rape;*" *"HIV can be prevented after rape;*" "*Is there a treatment that can be used to prevent HIV infection after someone has been raped?*". During analyses these were explored independently. Participants who knew that there was a treatment to prevent HIV after rape were asked to provide the name of the treatment. This was an open ended question. Responses were grouped thematically. Participants who knew there is a treatment to prevent HIV after rape were also asked where a person can go to get this treatment. Responses were not provided to the participant but were classified in the following categories: *Police;* *Public hospital, health centre or clinic*; *private hospital health centre or clinic*; *traditional healer; elsewhere. Those who knew there was a treatment were also asked (yes/no) if there was a time limit in which you need to take the treatment and then the time limit (open ended).* |
| **Sexual violence by a non-partner** | | For each of the following people, participants were asked to indicate (yes/no) whether the person had forced them to have sex or perform sex sexual acts that they did not want to a) Before the age of 15 years and b) after the age of sixteen years: Father; Stepfather; Another family member; teacher; police officer or soldier; boyfriends; stranger; someone from work; priest or religious leader; anyone else. These variables were combined as a count and also combined to create a dichotomous variable (experienced sexual violence from at least one non-partner). The later was used as the primary outcome of the multivariable analysis. For each person from whom the participant experienced sexual violence they were also asked to indicate the frequency: "Once or twice;" "A few times;" "Many times." This was used to determine a proxy for incidence as described in the main body of the paper. |
